# Small vessel dysfunction at 7T MRI locally predicts white matter damage progression in CADASIL

**DOI:** 10.1177/0271678X251369257

**Published:** 2025-08-20

**Authors:** Stanley DT Pham, Hilde van den Brink, Anna Kopczak, Naomi Vlegels, Alberto De Luca, Benno Gesierich, Michael S Stringer, Michael J Thrippleton, Joanna M Wardlaw, Alex A Bhogal, Nikki Dieleman, Jacobus JM Zwanenburg, Marco Duering, Geert Jan Biessels, Jeroen CW Siero

**Affiliations:** 1 Translational Neuroimaging Group, Center for Image Sciences, University Medical Center Utrecht, Utrecht University, the Netherlands; 2 Department of Neurology and Neurosurgery, UMC Utrecht Brain Center, University Medical Center Utrecht, Utrecht University, the Netherlands; 3 J. Philip Kistler Stroke Research Center, Department of Neurology, 2348 Massachusetts General Hospital, Harvard Medical School, Boston, MA, USA; 4 Institute for Stroke and Dementia Research, Klinikum der Universität München, Ludwig-Maximilians-Universität, Munich, Germany; 5 Image Sciences Institute, University Medical Center Utrecht, Utrecht University, Utrecht, The Netherlands; 6 Medical Image Analysis Center (MIAC), University of Basel, Basel, Switzerland; 7 Brain Research Imaging Center, Center for Clinical Brain Sciences, UK Dementia Institute Center at the University of Edinburgh, Edinburgh, UK; 8 Spinoza Centre for Neuroimaging, Amsterdam, Netherlands

**Keywords:** Small vessel disease, cerebrovascular reactivity, MRI, white matter damage, CADASIL

## Abstract

Cerebral small vessel diseases (cSVDs) contribute significantly to stroke and dementia. Advanced 7 T MRI techniques have revealed small vessel dysfunction in cSVD patients, linked to global white matter damage cross-sectionally. However, it remains unclear whether these vascular deficits predict progressive tissue damage. This longitudinal study examined the spatial relationship between local vascular function and white matter damage progression in patients with Cerebral Autosomal Dominant Arteriopathy with Subcortical Infarcts and Leukoencephalopathy (CADASIL). Twenty-two patients underwent baseline small vessel function assessment using 7 T MRI. Voxelwise blood-oxygenation level-dependent cerebrovascular reactivity (BOLD-CVR) to a hypercapnic was evaluated. White matter changes were assessed on 3 T MRI over two years, analyzing mean diffusivity changes and conversion of normal-appearing white matter to white matter hyperintensities (WMH). Results showed significant global increases in white matter damage over time. Voxelwise analysis revealed that lower baseline BOLD-CVR magnitude and higher dispersion were associated with increased white matter damage and WMH progression at specific locations at follow-up. However, whole-brain vascular function measures did not predict white matter changes at a global level. These findings suggest that local vascular function plays a key role in white matter damage progression in CADASIL, highlighting the importance of regional vascular health in cSVDs.

## Introduction

Cerebral small vessel diseases (cSVDs) affect the small arteries, capillaries, and veins in the brain and are a major cause of stroke and dementia.^1–3^ With brain MRI, cSVDs are mostly studied through markers of tissue damage, such as white matter hyperintensities (WMH), lacunes, microbleeds, or enlarged perivascular spaces.^4–6^ Complementing these markers with measures of small vessel function can offer insights into potential disease mechanisms at the microvascular level and support the development of much-needed treatments. Recently, we found in patients with Cerebral Autosomal Dominant Arteriopathy with Subcortical Infarcts and Leukoencephalopathy (CADASIL), a monogenic form of cSVD with a high burden of white matter damage, that multiple aspects of small vessel function, assessed with 7 T MRI, were affected compared to healthy controls.^7,8^ This included perforating artery blood flow pulsatility, indicative of vascular stiffness, and microvascular cerebrovascular reactivity (CVR) to a hypercapnic stimulus, indicative of endothelium-independent vascular reactivity. Abnormalities of small vessel function were also observed in patients with sporadic cSVD, with slightly different patterns of affected vessels, possibly reflecting different underlying processes.^9^ Both in CADASIL and sporadic cSVD, impaired small vessel function was also associated with global WMH burden.^7,9^

There are still open questions on how small vessel dysfunction contributes to the development of white matter damage in cSVDs. It has been noted previously that vascular reactivity and perfusion are worse in WMH than in normal-appearing white matter (NAWM).^8,10–12^ There are also indications that vascular function, in particular CVR across the NAWM, predicts total WMH volume growth over time.^13–16^ The question is, however, if these observations are reflective of poor global vascular health across the brain being associated with a higher risk of further tissue damage, or whether the actual local condition of the vessels predicts damage locally. Such local processes may help to understand why WMH occur at particular locations and why, also in the NAWM, there is local variance in injury severity.^17^

Therefore, this longitudinal study aims to determine the spatial coherence between local small vessel function and progression of white matter damage in patients with CADASIL. Our primary analyses assessed this association locally, at the voxel level, using functional measures of white matter (WM) CVR to a hypercapnic stimulus to assess local small vessel function. The WM CVR signal is generally low at clinical field strengths and may not be enough to derive meaningful local information. In this work, we therefore used 7 T MRI to assess WM CVR at the voxel level and associated it with incident WMH and deterioration of white matter microstructure measured with diffusion MRI (mean diffusivity, MD), two complementary, but distinct markers of progressive white matter damage. To put local voxel-wise associations in perspective to global effects, we performed secondary analyses with global small vessel function measures, including perforating artery flow and pulsatility in relation to global indicators of white matter damage.

## Materials and methods

### Study participants

Patients with CADASIL were recruited through the ZOOM@SVDs study, a prospective cohort study with a follow-up measurement after two years.^18^ This was a collaborative study between the University Medical Center Utrecht (UMCU) in the Netherlands and the Institute for Stroke and Dementia Research at Ludwig-Maximilians-Universität (LMU), Munich, Germany. At the LMU, 23 patients with CADASIL and 13 unrelated, age, and sex-matched controls were recruited for the baseline assessment between October 2017 and July 2019. Participants underwent a 3 T brain MRI at baseline and travelled to the UMCU 8–28 days after the baseline visit to undergo a 7T brain MRI. Not all participants successfully underwent all 7 T MRI scans. Follow-up MRIs were performed two years after baseline at LMU.

The present longitudinal study only included the CADASIL patients in the analysis. CADASIL was confirmed in these participants by molecular testing for mutation of the NOTCH3 gene or through skin biopsy. The Medical Ethics Review Committees of the UMCU (Project number NL62090.041.17) and LMU (Project Number 17-088) approved the study, which was conducted in accordance with the Declaration of Helsinki and the European law of General Data Protection Regulation. Written informed consent was obtained from all participants.

### MRI acquisition

All 3 T brain MRIs were performed at the LMU on a Siemens Magnetom Skyra 3T scanner (Siemens Healthineers, Erlangen, Germany) with a 64-channel head/neck coil. The scan protocol and sequence parameters have been published before and included a 3D T1-weighted gradient echo, a 3D fluid-attenuated inversion recovery (FLAIR), and a diffusion-weighted MRI scan.^18^

Participants underwent the 7T brain MRIs at the UMCU on a Philips scanner (Philips Healthcare, Best, The Netherlands) using a 32-channel receive head coil with a quadrature transmit coil (Nova Medical, MA, USA). The scan protocol and acquisition details have been published elsewhere and included a two-dimensional phase contrast (2 D-PC) sequence to assess blood flow velocity in perforating arteries at different locations in the vascular tree and blood oxygenation-level dependent (BOLD) sequences to assess vascular reactivity to both visual and hypercapnic stimuli.^18^

### WMH and brain volumetrics

WMH were segmented on bias-corrected T1-weighted and FLAIR scans using a modified 3 D U-Net deep learning algorithm, as described previously.^19^ This deep learning model was previously validated in CADASIL patients.^20^ The segmented WMH masks were manually checked and edited in case of misclassifications. All edits to WMH masks were performed while blinded to the BOLD-CVR values. Intracranial volume, grey matter (GM), and WM masks were automatically segmented from tissue probability maps, using a threshold of 0.5 with the Computational Anatomy Toolbox (CAT12).^18^

### Diffusion metrics

Diffusion-weighted imaging (DWI) data were processed following a previously described method.^21^ In short, the raw data were initially visually inspected for major artefacts. Scans were denoised and corrected for Gibbs, distortion, and motion artefacts and intensity bias. After pre-processing, the diffusion tensors were calculated on the b = 0 and b = 1000 m/s^2^ data to obtain the white matter MD maps. Peak width of skeletonised mean diffusivity (PSMD) was computed with the MD maps using the publicly available script (https://github.com/miac-research/psmd).^22^ PSMD is an index of the dispersion of the MD values across the white matter skeleton and has been described in detail before. PSMD_NAWM_ was computed for the NAWM. The NAWM masks were acquired by subtracting lesions (i.e. WMH and lacunes) from the total white matter mask.

### Small vessel function

Small vessel function was assessed on 7T MRI with two complementary measures in different regions of interest. All these measures, along with their respective analysis pipelines, have been described in detail before.^18^ In short, the following functional measures were assessed.

1. Small vessel CVR to a hypercapnic stimulus (breathing 6% CO_2_ in room air) was assessed using the BOLD time series acquired over the whole brain. Our processing pipeline included FSL MCFLIRT^23^ to correct for motion, boundary-based 3D smoothing with a 3 × 3 voxel kernel using FSL SUSAN^24^ to mitigate noise, and ICA-AROMA^25^ to identify motion-related components and remove them from the data. All components identified by ICA-AROMA were manually verified for artefacts related to high-frequency noise or due to the spatial location (e.g. sagittal sinus). For all the details on the pipeline used, refer to the supplementary table from our previous work.^7^ CVR was assessed voxel-wise (2 mm isotropic voxels) using a general linear model with the end-tidal CO_2_ (etCO_2_) trace as regressor. The CVR magnitude was reported as the BOLD% signal change per mmHg CO_2_, correcting for the ΔetCO_2_, computed as the difference between the average of the highest 25% and lowest 25% of the etCO_2_ trace, during the hypercapnic challenge.^26^ For the voxel-wise analyses, the ΔetCO_2_ was used as a covariate. In addition to the magnitude, we computed a CVR ‘dispersion’ related metric using the ‘fitTau.m’ function in the seeVR toolbox,^27^ which reflects the vascular response’s temporal blurring, or smearing, with a time constant τ. The temporal aspect of the CVR response is affected by vascular wall properties and blood redistribution effects, that are not reflected by CVR magnitude alone as that primarily reflects vascular dilatory capacity. Furthermore, studies have shown that dispersion, or lag, is altered in various cerebrovascular conditions.^14,28^ The dispersion time constant is calculated using a least squares fit between the voxel-wise BOLD response with a convolution of the end-tidal CO2 trace and an exponential function.^29^
2. Blood flow velocity and velocity pulsatility index in perforating arterioles at the level of the basal ganglia (BG) and semioval centre (CSO) using 2D-PC MRI to assess perforating arteriole stiffness, was analysed using an in-house developed tool for small vessel MRI markers (SELMA).^30^

### Local small vessel function and white matter damage progression

**Figure 1.**
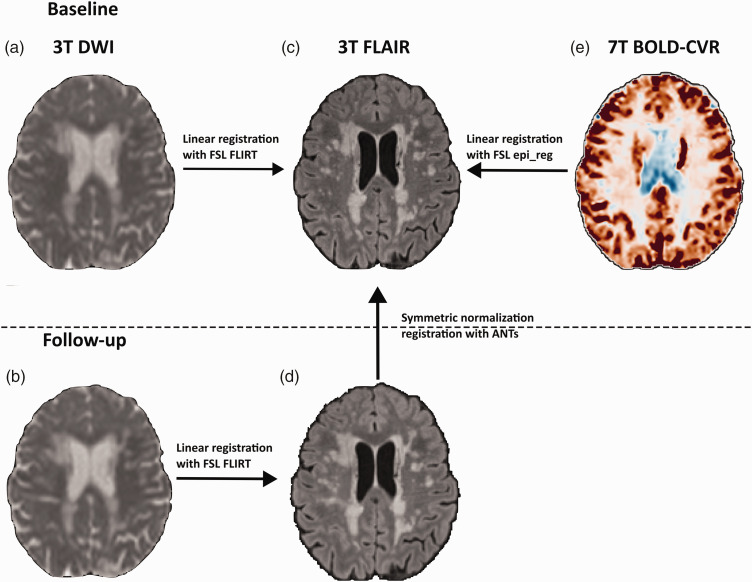
Registration-overview linking longitudinal white matter damage to baseline small vessel function. (a) DWI scans were registered to FLAIR scans at baseline and follow-up (b) using linear affine registration with ‘FSL FLIRT.^12^’ (d) Follow-up FLAIR scans were then registered to baseline FLAIR (c) using non-linear deformation with the symmetric normalisation method of the ANTs software package.^13,14^ (e) The functional 7T BOLD-CVR data (acquired voxel size 2 mm isotropic; upsampled to 1mm isotropic) were registered to baseline FLAIR data (voxel size 1 mm isotropic) using linear registration with ‘FSL epi_reg^12^’ and trilinear interpolation. ANTs: advanced normalization tools; BOLD: blood-oxygenation level-dependent; CVR: cerebrovascular reactivity; DWI: diffusion-weighted imaging; FLAIR: fluid-attenuated inversion recovery; FSL: FMRIB software library; MD: mean diffusivity; NAWM: normal-appearing white matter; WMH: white matter hyperintensity.

To quantify the progression of white matter damage over time locally, MD maps were first linearly registered to the FLAIR scans at baseline and follow-up, using FLIRT from the Functional Magnetic Resonance Imaging of the Brain (FMRIB) software library (FSL) v6.0.6.^31^ Follow-up FLAIR scans were then registered to the baseline FLAIR scans using a combination of affine registration and non-linear deformation with the symmetrical normalisation approach^32^ using the Advanced Normalization Tools (ANTs) software package.^33^ WMH were masked out during the deformation to prevent size changes of the lesions. Finally, 7T BOLD scans were registered to baseline FLAIR space using ‘epi_reg’ from FSL.^31^ All local analyses were thus performed in FLAIR space with 1 mm isotropic resolution. BOLD-CVR data were upsampled with trilinear interpolation to match the 1 mm isotropic resolution of the FLAIR data. An overview of the registration process is shown in Figure 1.

**Figure 2.**
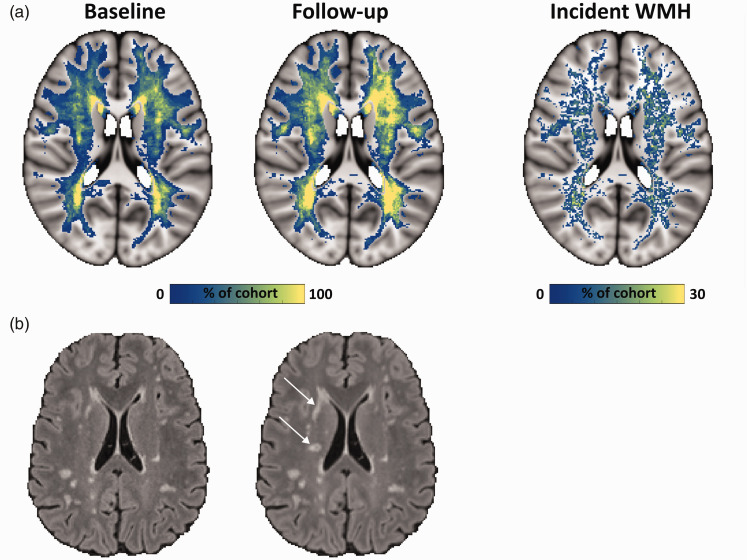
White matter hyperintensity changes after a two-year follow-up. (a) The top row shows a frequency map of WMH of the whole cohort at baseline and after two-year follow-up, including a frequency map of the locations with incident WMH and (b) FLAIR scans of an individual participant are shown in the bottom row for baseline on the left and follow-up in the middle. The arrows indicate new WMH lesions appearing after two-year follow-up. BOLD-CVR: blood-oxygenation level-dependent cerebrovascular reactivity; FLAIR: fluid-attenuated inversion recovery; GM: gray matter; NAWM: normal-appearing white matter; WMH: white matter hyperintensity; WM: white matter.

Following registration, the WM masks were eroded to correct for segmentation and registration errors at the boundary between WM and GM and to mitigate partial volume effects from GM. An erosion kernel of 2 mm was chosen based on the acquired resolution of the 7T BOLD-CVR data and the biological point-spread function of the BOLD signal (∼1.5 mm).^34^ Additionally, a region dilated 6 mm around the ventricles in FLAIR space was masked out to exclude the effects of the large subependymal veins around the ventricles on the BOLD-CVR data. The size of this region was empirically determined by choosing a dilation size that would mask out any remaining partial volume effects of the ventricles in all participants. Baseline WM MD maps were subtracted from follow-up WM MD maps in baseline FLAIR image space to obtain voxel-wise ΔMD maps. Baseline and follow-up WMH masks in baseline FLAIR image space were subtracted from each other in native FLAIR space to obtain masks indicating the stable voxels in NAWM and masks of the NAWM voxels that progressed to WMH, and similarly, masks of WMH voxels that reverted to NAWM after a two-year follow-up (Figure 2). We used these masks to define the “tissue fate” for all WM voxels, i.e., NAWM remaining stable, NAWM progressing to WMH, and WMH reverting to NAWM after a two-year follow-up.

### Global small vessel function and white matter damage progression

Global white matter injury progression was characterised as changes in total WMH volume and PSMD_NAWM_, measured in native FLAIR and DWI space, respectively, after a two-year follow-up. WMH at baseline and follow-up were normalised to intracranial volume (ICV). Normalised WMH and PSMD_NAWM_ were subsequently cube-root transformed. Global white matter damage progression was defined as follow-up WMH and PSMD_NAWM_ minus baseline WMH and PSMD_NAWM_ (i.e. ΔWMH = WMH_follow-up_ – WMH_baseline_; ΔPSMD_NAWM_ = PSMD_NAWM follow-up_ – PSMD_NAWM baseline_).

## Statistical analyses

### Descriptive analyses

First, the baseline mean BOLD-CVR magnitude and dispersion were compared between the NAWM and WMH with a paired t-test. Moreover, the global association between small vessel function and white matter damage burden at baseline was assessed with Spearman correlations because of the skewness of the baseline data. Correction for age was not performed because age is strongly collinear with disease burden in CADASIL.

Then, the median percentages of NAWM voxels progressing to WMH and WMH reverting to NAWM were computed across participants, with corresponding interquartile ranges (IQR). Last, a paired t-test was performed to assess if the changes in WMH volume and ΔMD_NAWM_ (ΔMD_NAWM_ =MD_NAWM follow-up_–MD_NAWM baseline_) and PSMD_NAWM_ after a two-year follow-up were significantly different.

### Primary voxel-wise analyses

To assess the voxel-wise associations of small vessel function measures and local progression of white matter damage across the whole cohort, all baseline NAWM voxels of all participants were concatenated into a single dataset with corresponding baseline BOLD-CVR magnitude, BOLD-CVR dispersion, ΔetCO_2_, tissue fate, ΔMD, and participant ID. Voxel-wise CVR magnitude (%) or dispersion (s) measurements were entered as individual fixed effects into linear mixed models for ΔMD and tissue fate, respectively. All models were corrected for ΔetCO2 as fixed effect. All models included the participant ID as a random effect to account for between-subject effects. Additionally, to accommodate for possible between-subjects age effects, we added Age as an additional fixed effect in the models in sensitivity analyses. These analyses were also performed separately for stable NAWM and NAWM to WMH voxels.

For “tissue fate”, linear mixed models were used, and odds ratios (OR) were calculated for the association between CVR magnitude and dispersion with the odds of NAWM progressing to WMH and the reversion of WMH to NAWM.

As an additional exploratory analysis, a one-way ANOVA was performed to compare the mean regional BOLD-CVR (magnitude, dispersion) at baseline between the white matter fate regions (stable NAWM, NAWM to WMH, and stable WMH) across all patients. Tukey’s HSD Test for multiple comparisons was used in the post-hoc analyses. We also report the BOLD-CVR in stable WMH and grey matter for comparison.

### Secondary global analyses

The global association between small vessel function at baseline and progressive white matter damage was assessed with regression analyses. All small vessel function measures were entered in univariate linear regression models with ΔWMH and ΔPSMD_NAWM_ as dependent variables.

All linear mixed model analyses were performed in R (version 4.2.1, R Foundation for Statistical Computing, Vienna, Austria) with the ‘lme4’ package. The exploratory and global analyses were performed in MATLAB (version R2021a, the MathWorks, Natick, MA). A significance level of p < 0.05 was considered significant after correction for multiple comparisons for all the primary, exploratory, and global secondary analyses.

### Data availability

The data that support the findings of this study are available upon reasonable request to the corresponding author.

## Results

Of the original 23 patients with CADASIL recruited at baseline, 22 had a successful follow-up. Seventeen of the 22 participants with a successful follow-up had a completed BOLD-CVR at baseline. The mean age of the 22 included patients was 51 years (range: 28–67). Six of the patients (27%) had a history of stroke. The median WMH volume was 51 mL, or 3.5% of ICV. Twelve patients (55%) had lacunes, and twelve patients had microbleeds detected with 3T MRI. Characteristics of the included patients have been described in detail before^7^ and are shown in Table 1. The median follow-up time of the study population was 25 months (range: 24–39 months).

**Table 1.** Baseline characteristics.

Demographics	
Number of participants	22
Age, years mean ± SD	51 ± 10
Female sex, n (%)	12 (55%)
Vascular risk profile	
History of stroke, n (%)	6 (27%)
Hypertension, n (%)	3 (14%)
Diabetes, n (%)	0 (0%)
Current/ever smoker, n (%)	15 (68%)
Baseline SVD burden	
WMH volume, % of ICV median [IQR]	3.5 [3.6]
Lacunes, n (%)	12 (55%)
Microbleeds, n (%)	12 (55%)
MD, mm^2^/s × 10^−4^ mean ± SD	6.28 ± 0.40
PSMD, mm^2^/s × 10^−4^ mean ± SD	3.13 ± 0.62

ICV: intracranial volume; IQR: inter-quartile range; MD: mean diffusivity; PSMD: Peak width of the skeletonized mean diffusivity; SD: standard deviation; WMH: white matter hyperintensities.

At baseline, the BOLD-CVR magnitude (n = 17) was lower in the WMH compared to NAWM (NAWM. mean ± SD: 0.054 ± 0.047%/mmHg vs. WMH: 0.013 ± 0.026%/mmHg; p < 0.001), and the dispersion was higher in WMH compared to NAWM (NAWM: 46 s ± 13s vs. WMH: 59 ± 15 s; p < 0.001). The BOLD-CVR magnitude in GM was 0.26 ± 0.13%/mmHg, and dispersion was 14 ± 8 s. Perforating artery pulsatility measures were obtained from all patients at baseline (Supplementary Fig. 1). The cross-sectional associations of global small vessel function measures with baseline white matter damage reported previously are in Supplementary Table 2, for reference. Blood flow velocity of the perforating arteries at the CSO was negatively associated with baseline WMH volume (Spearman’s ρ: −0.48; *p = *0.025) and PSMD_NAWM_ (Spearman’s ρ: −0.44; *p = *0.040). The pulsatility index of the perforating arteries at the CSO was negatively associated with baseline WMH volume (Spearman’s ρ: −0.43; *p = *0.049).

At follow-up, the median WMH volume increase [IQR] was 5.1 [8.7] mL (0.3 [0.6]% of ICV; p < 0.001) over all included patients (Table 2). The median volume [IQR] of NAWM voxels converting to WMH was 5.1 [8.3] mL (4.1 [8.3]% of total NAWM), and the median volume [IQR] of WMH voxels converting to NAWM was 0.15 [0.53] mL (1.3 [1.9]% of total WMH). There was a significant increase of ΔMD_NAWM_ (mean difference ± SD (mm^2^/s × 10^−4^): 0.13 ± 0.17; p = 0.003) after a two-year follow-up. ΔPSMD_NAWM_ also significantly increased after two-year follow-up (mean difference ± SD (mm^2^/s × 10^−4^): 0.15 ± 0.28; p = 0.011) (Supplementary Figure 2). Sensitivity analyses with Age as an additional covariate in the voxel-wise analyses did not show any differences in the results (Supplemental Material).

**Table 2.** Longitudinal changes of white matter damage.

	N = 22
ΔWMH volume, mL median [IQR]	5.1 [8.3]
ΔWMH volume, % of ICV median [IQR]	0.3 [0.6]
NAWM ΔMD, mm^2^/s × 10^−4^ mean ± SD	0.13 ± 0.17
NAWM ΔPSMD, mm^2^/s × 10^−4^ mean ± SD	0.15 ± 0.28

ICV: intracranial volume; IQR: inter-quartile range; MD: mean diffusivity; NAWM: normal appearing white matter; PSMD: Peak width of the skeletonised mean diffusivity; SD: standard deviation; WMH: white matter hyperintensities; in the 17 patients included in the BOLD-CVR analyses these numbers were similar: (Supplementary Table 1).

### Primary analyses

**Figure 3.**
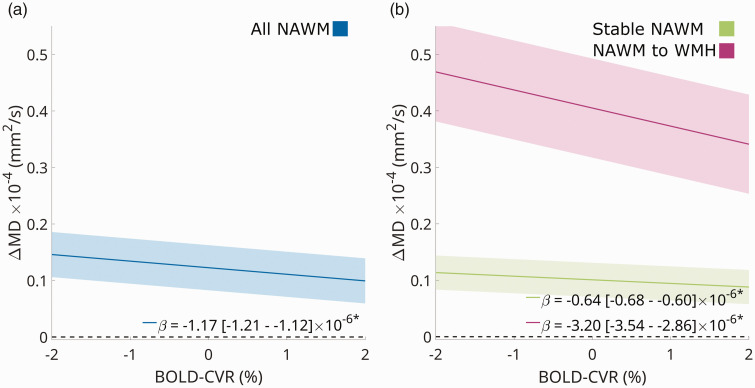
Voxelwise association between baseline BOLD-CVR and longitudinal ΔMD. Voxelwise linear mixed model associations between baseline BOLD-CVR and longitudinal ΔMD are shown for NAWM in FLAIR space (voxel size 1 mm isotropic). Associations are shown for all NAWM at baseline (a), separated into NAWM that remained stable (green) and progressed to WMH (purple) after a two-year follow-up (b). Unstandardised regression coefficients (β) are shown for each association with 95%-CI; *: all were significant at p < 1.0 × 10^−15^. The shaded area represents the standard error from the regression fit. BOLD: blood-oxygenation level-dependent; CI: confidence interval; CVR: cerebrovascular reactivity; MD: mean diffusivity; NAWM: normal-appearing white matter.

Baseline NAWM BOLD-CVR magnitude was negatively associated with baseline MD at voxel-level (β [95% CI]: −13.4 [−13.5 – −13.4] × 10^−6^; *p < *1.0 ×10^−15^), indicating that voxels with a lower baseline BOLD-CVR magnitude were associated with a higher baseline MD (Supplementary Fig. 1). For the primary longitudinal voxel-wise analysis, baseline NAWM BOLD-CVR magnitude was negatively associated with the change in MD (ΔMD) (β [95% CI]: −1.17 [−1.21 – −1.12] × 10^−6^; *p < *1 × 10^−15^) as shown in Figure 3. This negative association was observed both in NAWM voxels remaining stable and in those converting to WMH, albeit five-fold stronger in the latter (NAWM stable: β [95% CI] = −0.64 [−0.68–−0.60] ×10^−6^; *p < *1 × 10^−15^, NAWM to WMH: β [95% CI] = −3.20 [−3.54–−2.86] × 10^−6^; *p < *1 × 10^−15^).

Similarly, BOLD-CVR dispersion at baseline was positively associated with baseline MD at voxel-level (β [95% CI]: 0.30 [0.30–0.31]×10^−6^; *p < *1 × 10^−15^). Longitudinally, the BOLD-CVR dispersion was positively associated with ΔMD on a voxel-level (β [95% CI]: 0.047 [0.045–0.049]×10^−6^; *p < *1 × 10^−15^). This positive association between BOLD-CVR dispersion and ΔMD was observed both in stable NAWM voxels and those converting to WMH, with the latter association being three times stronger (NAWM stable: β [95% CI] = 0.034 [0.032–0.036]×10^−6^; *p < *1 × 10^−15^, NAWM to WMH: β [95% CI] = 0.11 [0.096–0.12]×10^−6^; *p < *1 × 10^−15^).

BOLD-CVR also predicted conversion of NAWM to WMH at voxel-level; the lower the CVR magnitude and the higher the BOLD-CVR dispersion, the higher the chance of conversion (OR per % 0.81 ([95% CI]: [0.81–0.82]; *p < *1 × 10^−15^ and per 10 s 1.06 ([95% CI]: [1.06–1.06]; *p < *1 × 10^−15^, respectively; Table 3). Moreover, BOLD-CVR magnitude was positively and dispersion negatively associated with the odds of WMH reverting back to NAWM (magnitude OR per % 1.06 ([95% CI]: [1.04–1.09]; *p = *1.3 × 10^−7^ and dispersion OR per 10 s 0.95 ([95% CI]: [0.95–0.96]; *p < *1 × 10^−15^, respectively; Table 3). Baseline BOLD-CVR according to tissue fate regions of interest are presented in Supplementary Table 1, for reference.

**Table 3.** Voxelwise associations between local BOLD-CVR at baseline and tissue fate.

	NAWM to WMH		WMH to NAWM	
	OR [95%-CI]	p-value	OR [95%-CI]	p-value
BOLD-CVR magnitude (%)	0.81 [0.81 – 0.82]	<1.0 × 10^−15^	1.06 [1.04 – 1.09]	1.3 × 10^−7^
BOLD-CVR dispersion (×10 s)	1.06 [1.06 – 1.06]	<1.0 × 10^−15^	0.95 [0.95 – 0.96]	<1.0 × 10^−15^

The linear mixed model results between BOLD-CVR in NAWM and voxelwise tissue fate are represented with ORs with 95%-CI and p-values. ORs are given per percentage of BOLD-CVR magnitude and per 10 s of BOLD-CVR dispersion. A p-value ≤0.05 denotes statistical significance.

BOLD: blood-oxygenation level-dependent; CI: confidence interval; CVR: cerebrovascular reactivity; NAWM: normal-appearing white matter; OR: odds ratio; WMH: white matter hyperintensity.

### Secondary global analyses

Table 4 shows the global associations of the baseline small vessel function measures with longitudinal white matter damage change. No consistent relations were found between any of the global small vessel function measures, including BOLD-CVR, and the progression of WMH volume or PSMD_NAWM_ after a two-year follow-up (all *p* > 0.05), also in terms of directions of effects.

**Table 4.** Associations between global small vessel function at baseline and global longitudinal white matter damage change.

	Global white matter damage change			
	ΔWMH volume		NAWM ΔPSMD	
Small vessel function at baseline	β [95% CI]	p	β [95% CI]	p
Pulsatility, CSO [N = 22]				
Blood flow velocity [cm/s]	0.09 [−0.35 – 0.54]	0.69	0.004 [−0.45 – 0.45]	0.99
Pulsatility Index ratio	0.09 [−0.36 – 0.53]	0.71	−0.01 [−0.46 – 0.44]	0.96
Pulsatility, BG [N = 22]				
BG Blood flow velocity [cm/s]	0.002 [−0.46 – 0.46]	0.99	0.11 [−0.35 – 0.57]	0.63
BG Pulsatility Index ratio	−0.21 [−0.67 – 0.24]	0.37	−0.12 [−0.58 – 0.34]	0.61
BOLD-CVR, NAWM [N = 17]				
BOLD-CVR magnitude [%]	−0.18 [−0.68 – 0.32]	0.49	−0.09 [0.59 – 0.41]	0.73
BOLD-CVR dispersion [s]	−0.22 [−0.72 – 0.27]	0.39	−0.17 [−0.67 – 0.33]	0.51

Baseline associations between global measures of small vessel function and white matter damage. For white matter damage, ΔWMH volume and NAWM ΔPSMD were used. p ≤ 0.05 denoted statistical significance. All BOLD-CVR models were adjusted for end-tidal CO_2_.

β: standardised beta; BG: basal ganglia; BOLD: blood-oxygenation level-dependent; CI: confidence interval; CSO: centrum semioval; NAWM: normal-appearing white matter; PSMD: Peak width of the skeletonized mean diffusivity; WMH: white matter hyperintensities.

## Discussion

Using high-field 7T MRI and voxel-wise analyses, we show that local white matter CVR predicts local progression of white matter damage over time, reflected in increasing MD values and progression of WMH, whereas higher CVR in WMH predicts reversal to NAWM in patients with CADASIL. The secondary global analysis did not capture an association between small vessel function and progression of white matter damage globally across the brain, likely due to a lack of sensitivity. These findings imply that local deficits in vascular function contribute to the development of white matter damage over time in patients with CADASIL.

Previous studies have reported an association between cerebrovascular function and white matter damage, primarily cross-sectionally and globally or at a region-of-interest level.^8,10,11^ A consistent finding among these studies was that vascular function (lower BOLD-CVR or CBF) was worse in WMH compared to NAWM, in accordance with our findings.^8,10–12,35^ Both reduced baseline CBF and a longitudinal decline in CBF were found to be associated with the development of WMH.^35,36^ Few published longitudinal studies found an association between worse CVR and WMH progression in CADASIL or elderly with moderate to severe WMH.^13–16^ A lower baseline CVR induced with acetazaolamide and assessed with phase-contrast MRI was found to be associated with a larger increase of WMH in CADASIL.^16^ Studies that assessed BOLD-CVR, specifically in NAWM regions progressing to WMH, found that magnitude and dispersion in those regions were worse than in NAWM which remained stable,^14,15^ in accordance with the findings of our primary voxel-wise analyses. Moreover, the negative voxel-wise association between BOLD-CVR and change in MD over time was observed across the NAWM, both in stable NAWM and NAWM that converted to WMH, albeit stronger in the latter. Hence, BOLD-CVR not only predicts the development of new lesions, it also informs on pre-lesional microstructural changes reflected by diffusion MRI. The lack of associations found in our secondary global analyses is in contrast with findings in literature and is likely caused by our limited sample size. One previous study investigated voxel-wise associations between 3T MRI BOLD-CVR and WMH presence.^37^ This was done cross-sectionally in patients with migraines, in whom voxels with a reduced CVR had a higher probability of having WMH. At 3T, however, voxel-wise analyses with functional data are challenging due to the limited resolution and diminished BOLD signal sensitivity in white matter due to the relatively low basal perfusion. To perform the voxel-wise analyses in our study, we utilised high-field 7T MRI, which offers higher inherent sensitivity and elevated BOLD contrast, enabling higher spatial resolution and thereby facilitating the capture of BOLD signals in small areas of white matter.^38^ Furthermore, at higher field strengths, the BOLD signal is more specific towards the microvasculature,^39^ which directly feeds the adjacent parenchyma, focusing the spotlight of the measurement more at the local condition of the microvasculature, which is more difficult to capture on clinical field strengths. In addition, our advanced processing pipeline included removing motion-related variance using independent-component analysis, removing non-specific large vessels, and mitigating partial volume effects between GM and WM.^7^ With this, we showed that focal lower CVR magnitude and greater dispersion/delay predict white matter damage progression, while focal higher CVR and lower dispersion predict the reversal of WMH, which may be missed by only studying the relation on a global level, especially in small studies. The finding is consistent with focal clusters of dilated vessels in WMH that are progressing to cavitation, also seen in CADASIL, and where CVR is focally very poor, reported previously in the INVESTIGATE@SVDs sister study in the SVDs@Target Programme.^40^

This study was performed in patients with CADASIL, a genetic disorder caused by a mutation of the NOTCH3 gene with a very distinct pathophysiology and rapid lesion growth. We found a WMH growth of 0.5% of ICV over two years, which aligns with what was previously reported for CADASIL.^41^ Interestingly, some WMH voxels reverted to NAWM after a two-year follow-up in all patients; however, the amount of regression observed in our study was lower than the threshold (0.25 mL) that has been proposed previously in literature as the minimum amount of regression that can be visually appreciated.^42,43^ Pre-clinical studies have shown that mice with the NOTCH3 mutation exhibit impaired cerebral blood flow regulation and impaired cerebrovascular function prior to structural abnormalities, such as brain parenchyma lesions or loss of vascular smooth muscle cells.^44,45^ Loss of smooth muscle cells is also reported at autopsy in patients with CADASIL.^46,47^ Of note is that CVR, in response to a hypercapnic stimulus, is predominantly a myogenic mechanism of the vascular smooth muscle cells. When impaired, this leads to decreased relaxation or increased resistance of the small vessels.^44^ Our findings show that impaired BOLD-CVR could be a marker for local dysfunction, possibly reflecting the loss of vascular smooth muscle cells, and an interesting intervention target for preventing disease progression in cSVDs. A recent study into the effects of antihypertensive drugs on CVR found that different classes of antihypertensive drugs differentially affect global CVR in patients with CADASIL.^48^ This shows that, despite the vascular pathology, CVR can still be manipulated in patients with CADASIL. The findings of our study warrant further research on CVR and how it could be influenced to prevent disease progression in cSVDs.

We demonstrated that locally impaired BOLD-CVR precedes the local progression of cSVDs. Still, how it interacts with other locally contributing factors of cSVDs, such as tissue perfusion,^49^ blood-brain-barrier integrity,^50^ cerebral autoregulatory reserve,^51^ or oxygen metabolism is unknown.^52^ With the use of more advanced MR or hybrid PET-MRI based imaging approaches, other contributing factors in cSVDs such as local oxygen extraction or glucose metabolism, can be captured and could complement BOLD-CVR by providing a more comprehensive understanding of tissue health and vascular-metabolic coupling.^53^ It is also unclear at what stage of the disease CVR becomes impaired, though it has been reported to happen in the early stages of neurodegenerative disorders.^54,55^ Likely, BOLD-CVR is not the only vascular function predictor of white matter progression, and other measures used in this study, reflecting small vessel pulsatility, could be associated on a local level as well. However, the nature of these other measurements does not support voxel-wise analyses. The relative importance of CVR, or small vessel function in general, as a predictor of progressive white matter damage in cSVDs needs to be further studied, potentially benefiting from AI-based prediction modelling,^56,57^ especially in combination with cardiovascular risk factors and other functional measures that can be obtained from different modalities. Such a multi-modal approach would better characterise the role of small vessel function in the progression of cSVDs.

### Strengths and limitations

The strengths of this study lie in its longitudinal design, with a homogeneous study population and advanced methods using 7T MRI to measure small vessel function on a voxel-wise level. The voxel-wise analyses using linear mixed models captured the subtle progression of two different complementary, yet distinct measures of white matter damage in relation to local deficits of BOLD-CVR, also owing to the large number of voxels that could be included across patients in the analyses.

This study also had several limitations. Fine-graded voxel-wise analyses may be affected by registration errors between modalities and between baseline and follow-up. This could lead to assigning the wrong tissue fate to WM voxels at baseline and may add variance to the voxel-wise analysis. Such registration errors likely have the biggest potential impact on the NAWM to WMH classification. However, these registration errors would lead to both under- and overestimations of the effect size. Moreover, we observed similar findings with the DWI data, suggesting that potential registration errors did not have a decisive effect on the results. Another potential limitation is the physiological variability of CVR across anatomical regions, which could influence the results. However, given that the association between baseline CVR and longitudinal damage was observed over the entire white matter irrespective of location, we believe our findings primarily reflect the disease-driven processes rather than region-specific difference in baseline CVR. Another limitation was the short follow-up time, relatively low disease burden, and sample size of our population, which might have affected the observable range of global damage progression and could possibly explain the lack of observed global effects, but much less so for the primary, voxel-based analyses. Lastly, the study was only performed in patients with CADASIL, meaning that these results do not necessarily generalise to other forms of cSVD, but as mentioned before, it does provide a homogenous study condition. This is further emphasised by previous work^7,9^ showing that different small vessel function measures are affected between CADASIL and sporadic cSVDs. Other studies have shown that vascular dysfunction in CADASIL and sporadic cSVDs are in different proportions relative to each other, reflecting different stages of the disease.^8^ Further research, including other forms of cSVDs, with larger populations and longer follow-up times, is warranted to compare with our results found in CADASIL.

## Conclusion

These results show that in patients with CADASIL, cerebrovascular reactivity predicts further white matter damage and reversal of WMH locally, at the voxel level, but not globally. These results suggest that local small vessel dysfunction is causally related to white matter damage progression.

## Supplemental Material

sj-pdf-1-jcb-10.1177_0271678X251369257 - Supplemental material for Small vessel dysfunction at 7T MRI locally predicts white matter damage progression in CADASIL Supplemental material, sj-pdf-1-jcb-10.1177_0271678X251369257 for Small vessel dysfunction at 7T MRI locally predicts white matter damage progression in CADASIL by Stanley DT Pham, Hilde van den Brink, Anna Kopczak, Naomi Vlegels, Alberto De Luca, Benno Gesierich, Michael S Stringer, Michael J Thrippleton, Joanna M Wardlaw, Alex A Bhogal, Nikki Dieleman, Jacobus JM Zwanenburg, Marco Duering, Geert Jan Biessels and Jeroen CW Siero in Journal of Cerebral Blood Flow & Metabolism
