# Supplementary material for: Small vessel dysfunction at 7T MRI locally predicts white matter damage progression in CADASIL
Source: J Cereb Blood Flow Metab. 2025 Aug 20;46(2):521–33. doi: 10.1177/0271678X251369257 (PMC12367715; doi:10.1177/0271678X251369257)
Supplement: Supplementary file 1 [file sj-pdf-1-jcb-10.1177_0271678X251369257.pdf]

Supplementary analyses to the results section

Participant flow chart

Supplementary Figure 1 shows the availability of the small vessel function measures included for all the analyses.

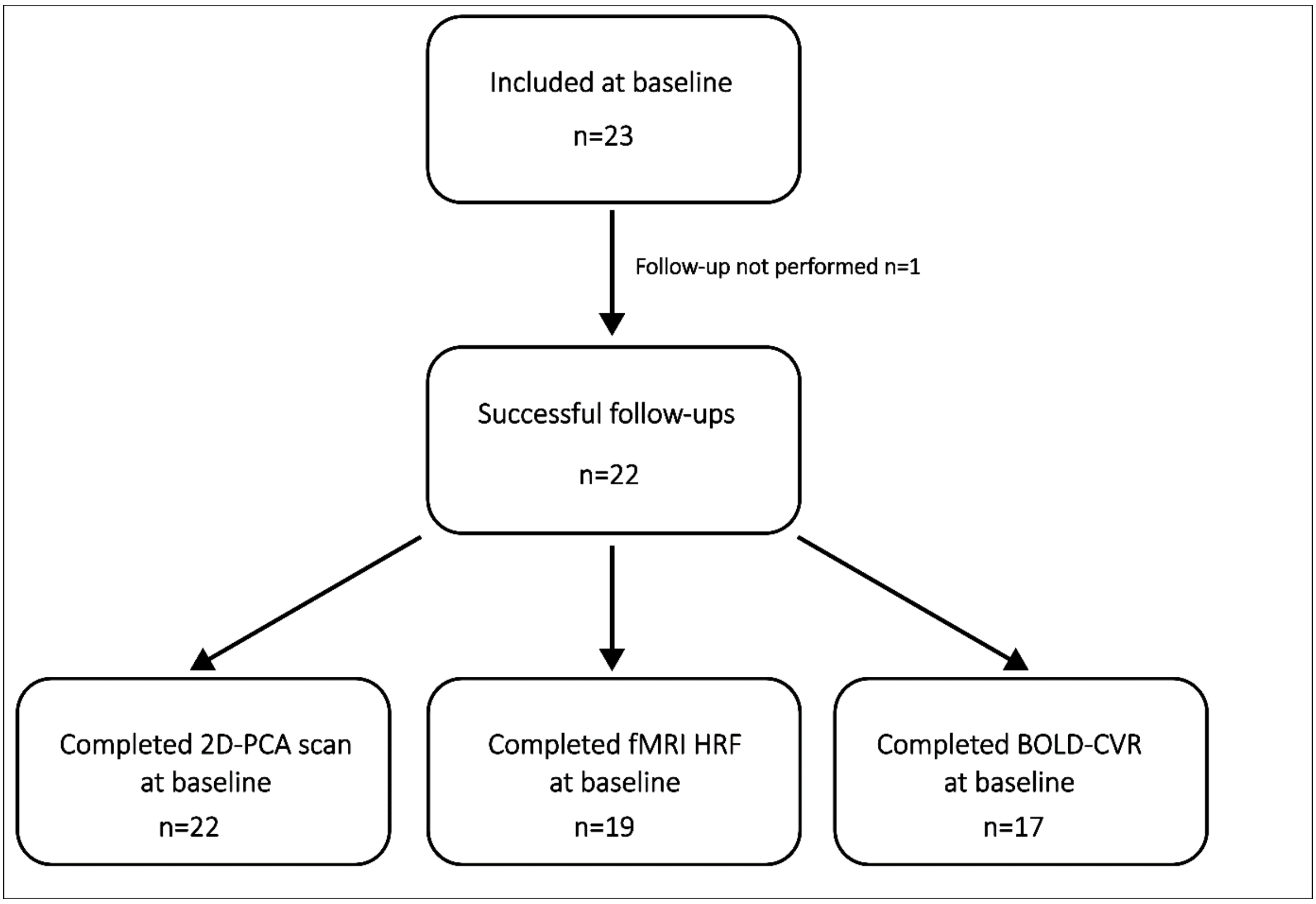

Supplementary Figure 1. Inclusion flow chart of CADASIL patients

Inclusion flow chart of CADASIL patients in the analyses. The voxel-wise analyses were performed on n=17 participants with available BOLD-CVR performed at baseline.

BOLD = blood-oxygenation level-dependent; CVR = cerebrovascular reactivity; fMRI = functional MRI; HRF = hemodynamic response function; PCA = phase-contrast angiography

Descriptive analyses

Supplementary Table 1 shows the white matter changes over time in the 17 patients with available CVR data, compared to the total group of 22. Lesion progression of the 17 patients was essentially the same as for the whole group.

Supplementary Table 1. Longitudinal changes of white matter damage

	**N=17**	**N=22**
ΔWMH volume, mL median [IQR]	5.1 [9.7]	5.1 [8.3]
ΔWMH volume, % of ICV median [IQR]	0.4 [0.6]	0.3 [0.6]
NAWM ΔMD, $mm^2/s \times 10^{-4}$ mean±SD	0.14±0.20	0.13±0.17
NAWM ΔPSMD, $mm^2/s \times 10^{-4}$ mean±SD	0.17±0.31	0.15±0.28

ICV = intracranial volume; IQR = inter-quartile range; MD = mean diffusivity; NAWM = normal appearing white matter; PSMD = Peak width of the skeletonized mean diffusivity; SD = standard deviation; WMH = white matter hyperintensities

Voxel-wise analyses at baseline

Supplementary Figure 1 shows the voxelwise association between NAWM BOLD-CVR magnitude and baseline MD. NAWM BOLD-CVR magnitude was negatively associated with baseline MD at voxel-level (β [95% CI]: -13.4 [-13.5 – -13.4]$\times 10^{-6}$; $p<1.0\times 10^{-15}$), indicating that voxels with a lower baseline BOLD-CVR magnitude were associated with a higher baseline MD.

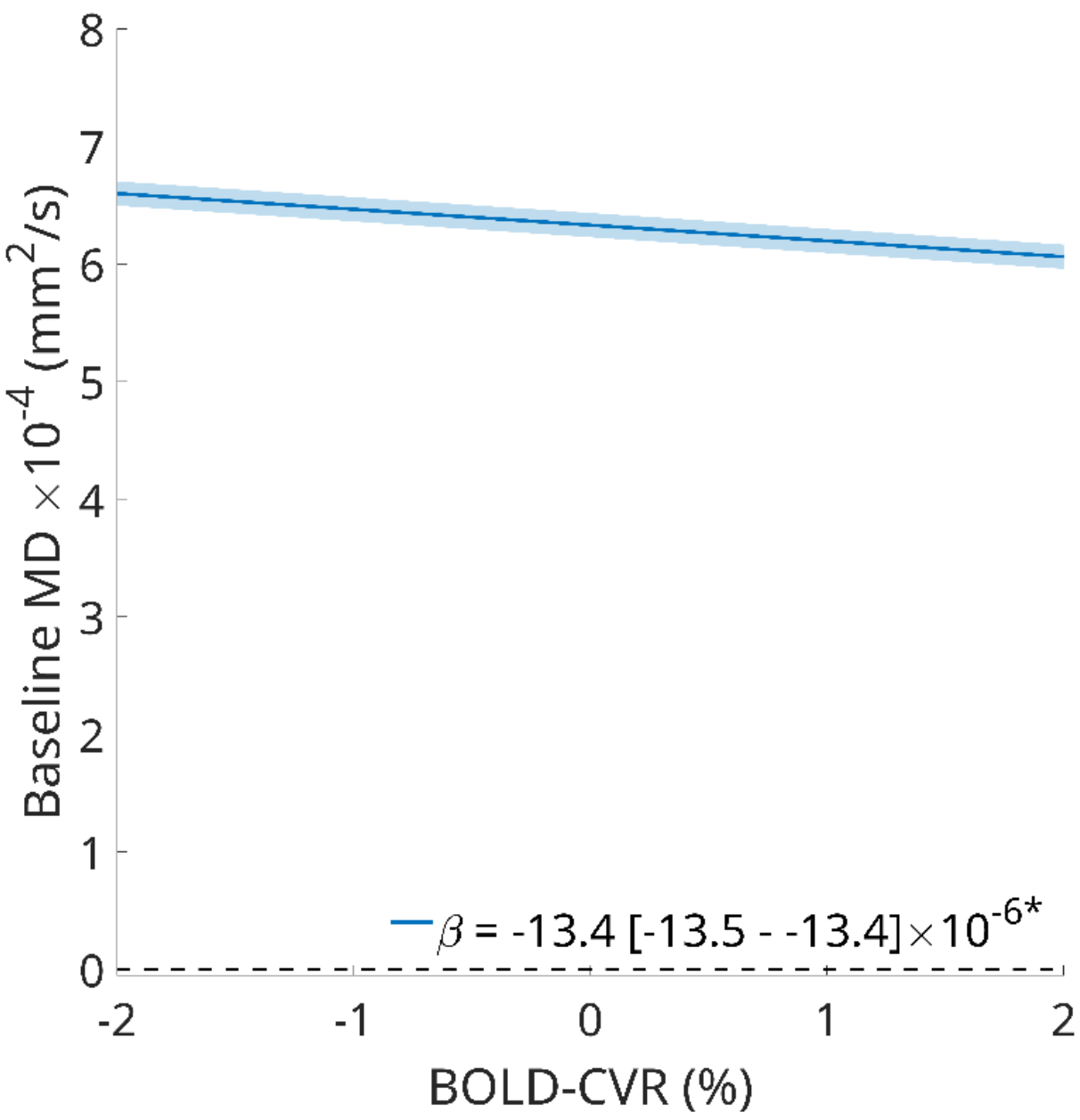

Supplementary Figure 2. Voxelwise association between BOLD-CVR and MD in NAWM at baseline

The voxelwise linear mixed model association between baseline BOLD-CVR and baseline MD is shown for all NAWM. The unstandardised regression coefficient (β) is demonstrated with 95%-CI and p-value. The shaded area represents the standard error from the regression fit. * denotes a p-value$<1.0\times 10^{-15}$

BOLD = blood-oxygenation level-dependent; CI = confidence interval; CVR = cerebrovascular reactivity; MD = mean diffusivity; NAWM = normal-appearing white matter

Exploratory longitudinal analyses

BOLD-CVR magnitude and dispersion were also compared according to tissue fate regions of interest (Supplementary Table 1). A one-way ANOVA revealed an overall statistically significant difference in BOLD-CVR magnitude across regions ($F(3,64) = 4.36$; $p=0.0074$). Post-hoc Tukey's HSD test showed a significant difference between stable NAWM regions (mean±SD (%): 0.87±0.61) versus stable WMH regions (mean±SD (%): 0.21±0.40; $p=0.0044$). The BOLD-CVR dispersion significantly differed between at least two regions ($F(3,64) = 3.2$; $p=0.03$). The dispersion was significantly lower in stable NAWM regions (mean±SD (s): 43±12) compared to stable WMH regions (mean±SD (s): 58±15; $p=0.02$).

Supplementary Table 2. BOLD-CVR at baseline according to tissue fate

	Primary regions of interest			
	**Stable NAWM**	**NAWM to WMH**	**WMH to NAWM**	**Stable WMH**
**BOLD-CVR magnitude** (%/mmHg)	0.056±0.048*	0.029±0.044	0.032±0.040	0.013±0.026*
**BOLD-CVR dispersion** (s)	43±12*	51±15	48±17	58±15*

Values are first calculated per patient and then averaged and expressed as mean±SD. BOLD-CVR magnitude ($F(3,64) = 4.36$; $p=0.0074$) and dispersion ($F(3,64) = 3.2$; $p=0.03$) significantly differed across the regions tested with a one-way ANOVA. * denotes statistically significant difference after post-hoc multiple comparison tests (Tukey's HSD).

BOLD = blood-oxygenation level-dependent; CVR = cerebrovascular reactivity; GM = gray matter; NAWM = normal-appearing white matter; SD = standard deviation; WMH = white matter hyperintensity

Secondary global analyses

There was a significant increase of global WMH volume (0.3% of ICV; p <0.001) and NAWM PSMD (0.15 $mm^2/s \times 10^{-4}$ p=0.011) after two-year follow-up in all included CADASIL patients. Supplementary Figure 2 shows the boxplots and all data points of the WMH volume and PSMD at baseline and follow-up. Supplementary Table 2 shows all the global associations between small vessel function and baseline WMH volume and baseline PSMD.

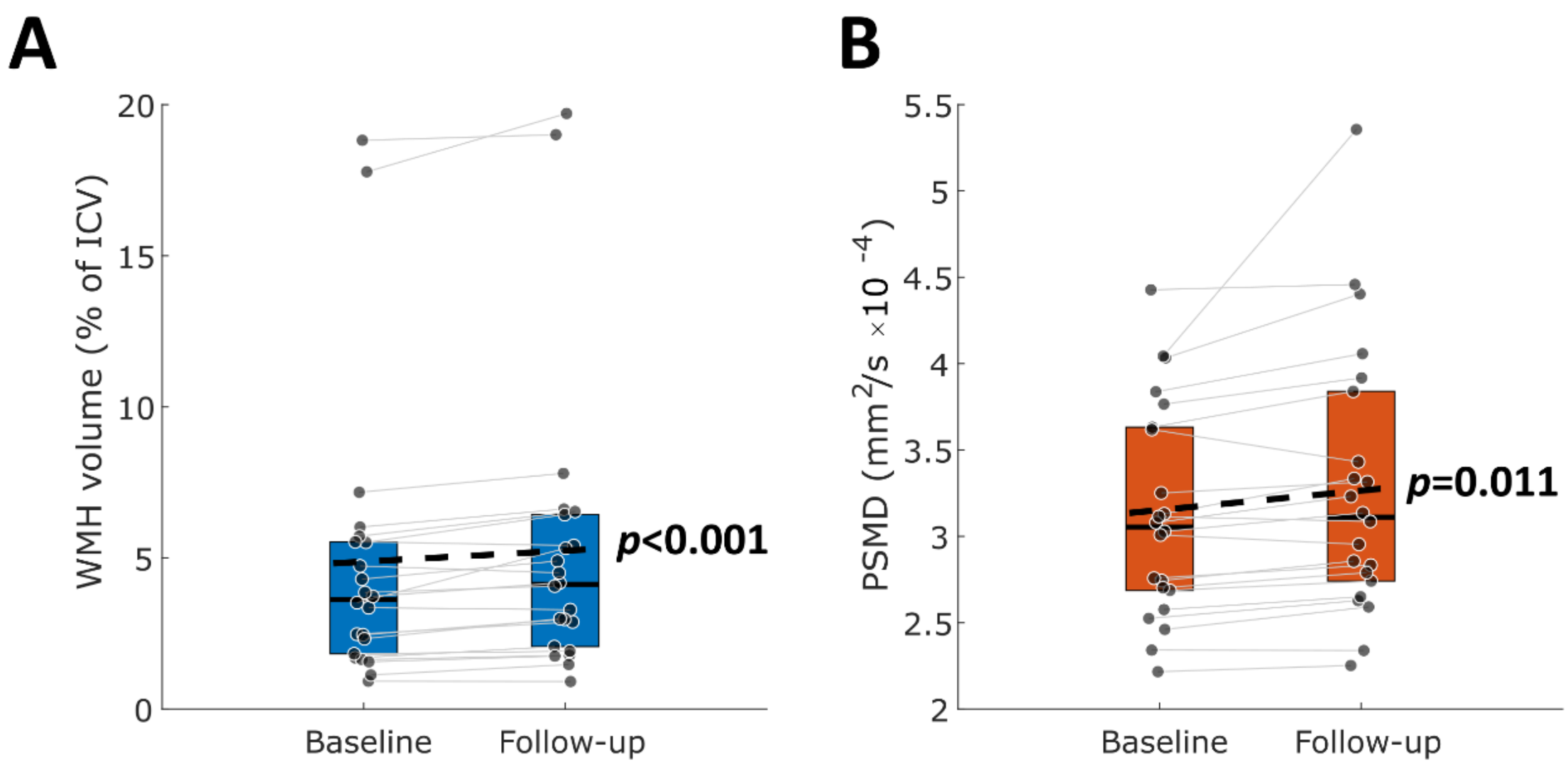

Supplementary Figure 3. Global white matter damage progression after a two-year follow-up

(A) Boxplots with median and 25th and 75th percentiles of WMH volume at baseline and after a two-year follow-up. WMH volume is expressed as a percentage of ICV. (B) Boxplots of the PSMD at baseline and follow-up. Lines represent the change of individual participants. WMHs and PSMD change were tested with a paired t-test where a p-value≤0.05 denotes statistical significance.

ICV = intracranial volume; PSMD = Peak width of the skeletonised mean diffusivity; WMH = white matter hyperintensities

Supplementary Table 2. Associations at baseline between small vessel function and global measures of white matter damage

	Baseline white matter damage			
**Small vessel function at baseline**	**WMH volume**		**PSMD**	
	**ρ**	**p-value**	**ρ**	**p-value**
**Pulsatility, NAWM [N = 22]**				
CSO Blood flow velocity [cm/s]	-0.48	**0.025**	-0.44	**0.040**
CSO Pulsatility index	-0.43	**0.049**	-0.23	0.295
BG Blood flow velocity [cm/s]	-0.29	0.194	-0.04	0.868
BG Pulsatility index	0.27	0.235	0.09	0.711
**BOLD short visual stimulus, GM [N = 19]**				
BOLD% signal change	-0.29	0.235	-0.42	0.077
Full width at half max [s]	0.29	0.229	0.20	0.410
**BOLD-CVR, NAWM [N = 17]**				
BOLD-CVR magnitude (%)	0.38	0.131	0.38	0.133
BOLD-CVR dispersion [s]	0.23	0.372	0.003	0.996

Baseline associations between global measures of small vessel function and white matter damage. For white matter damage, WMH volume and PSMD were used. p ≤0.05 denoted statistical significance. These baseline associations have been partially published elsewhere.[13]

BG = basal ganglia; BOLD = blood-oxygenation level-dependent; CSO = centrum semioval; PSMD = Peak width of the skeletonised mean diffusivity; ρ = Spearman's correlation coefficient; WMH = white matter hyperintensities

Sensitivity Analyses with Age

Models without age as covariate

	β delta MD in all NAWM	β delta MD in NAWM stable	β delta MD in NAWM to WMH
**BOLD-CVR magnitude** (%)	-1.17 [-1.21 – -1.12]×10^{-6}	-0.64 [-0.68 – -0.60]×10^{-6}	-3.20 [-3.54 – -2.86]×10^{-6}
**BOLD-CVR dispersion** (s)	0.047 [-0.47 – 1.37]×10^{-6}	0.034 [0.032 – 0.036]×10^{-6}	0.11 [0.096 – 0.12]×10^{-6}

	Odds Ratio NAWM to WMH	Odds Ratio WMH to NAWM
**BOLD-CVR magnitude** (%)	0.81 [0.81 – 0.82]	1.06 [1.04 – 1.09]
**BOLD-CVR dispersion** (s)	1.06 [1.06 – 1.06]	0.95 [0.95 – 0.96]

Models with age as covariate

	β delta MD in all NAWM	β delta MD in NAWM stable	β delta MD in NAWM to WMH
**BOLD-CVR magnitude** (%)	-1.17 [-1.21 – -1.12]×10^{-6}	-0.64 [-0.68 – -0.60]×10^{-6}	-3.20 [-3.54 – -2.86]×10^{-6}
**BOLD-CVR dispersion** (s)	0.047 [0.045 – 0.049]×10^{-6}	0.034 [0.031 – 0.036]×10^{-6}	0.11 [0.096 – 0.12]×10^{-6}
**Age (y)**	0.45 [-0.47 – 1.37]×10^{-6}	0.26 [-0.43 – 0.96]×10^{-6}	0.79 [-1.08 – 2.66]×10^{-6}

	Odds Ratio NAWM to WMH	Odds Ratio WMH to NAWM
**BOLD-CVR magnitude** (%)	0.81 [0.81 – 0.82]	1.06 [1.04 – 1.09]
**BOLD-CVR dispersion** (s)	1.06 [1.06 – 1.06]	0.95 [0.95 – 0.96]
**Age (y)**	1.02 [0.98 – 1.06]	0.96 [0.91 – 1.01]
